# Crystallization Kinetics of Polyamide 12 during Selective Laser Sintering

**DOI:** 10.3390/polym10020168

**Published:** 2018-02-09

**Authors:** Meng Zhao, Katrin Wudy, Dietmar Drummer

**Affiliations:** Institute of Polymer Technology (LKT), Friedrich-Alexander-University Erlangen-Nuremberg, 91054 Erlangen, Germany; zhao@lkt.uni-erlangen.de (M.Z.); wudy@lkt.uni-erlangen.de (K.W.)

**Keywords:** selective laser sintering, polyamide 12 (PA12), crystallization, differential scanning calorimeter

## Abstract

Selective laser sintering (SLS) of thermoplastic materials is an additive manufacturing process that overcomes the boundary between prototype construction and functional components. This technique also meets the requirements of traditional and established production processes. Crystallization behavior is one of the most critical properties during the cooling process and needs to be fully understood. Due to the huge influence of crystallization on the mechanical and thermal properties, it is important to investigate this process more closely. A commercial SLS polyamide (PA12) powder was measured with differential scanning calorimetry (DSC) to model a wider temperature range. To model isothermal crystallization between 160 and 168 °C, the Avrami model was used to determine the degree of crystallization. For non-isothermal crystallization between 0.2 and 20 K/min, different models were compared including the Ozawa, Jeziory, and Nakamura equations.

## 1. Introduction

Selective laser sintering (SLS) of polymers is established to produce prototypes and individualized parts. Selective laser sintering is a powder-based manufacturing process in which the components are built up layer by layer from a powder material using a laser. The overall process of SLS can be divided into the following three steps: powder application, energy input, and the consolidation phase [1].

- First, the building platform is lowered to a layer thickness of about 100 µm and the pre-heated powder is applied into the building chamber with a roller or blade.
- Second, the semi-crystalline thermoplastic powder is heated to a temperature just below the melting point. When the building chamber temperature is reached, depending on the given layer geometry, it connects to the individual layer below, and a CO_2_ laser, which is guided over the scanner mirror, selectively melts the preheated powder particles. The laser should only introduce the necessary amount of heat to melt a layer, to ensure the connection to the lower layer, and to minimize warpage during cooling.
- Last, in the subsequent consolidation phase, the melt is cooled again to the building chamber temperature; crystallization of the powder melt should not occur in this phase. This results in the formation of a two-phase mixing region in which the surrounding powder has approximately the same temperature as the molten powder. The surrounding powder, which is not melted, serves a support function and also serves as an insulating layer and is intended to ensure uniform cooling and thus homogeneous crystallization [2]. The described steps are repeated until the component is finished.

The commercial availability of polymer powders with good processability is limited: 95% of the total market share is made up of polyamide-based materials [3], of which polyamide 12 (PA12) is the most commonly used material.

SLS is understood as a quasi-isothermal process, which means a mixture of powder and melt at the same time during the building job [4], and the building and cooling process in SLS might take a few hours. Thermal properties of PA12 depend closely on the temperature and time [5,6]. The crystallization kinetic study is a fundamental study of the SLS component. With the crystallization analysis, the temperature–time-dependent material states are simulated during the building and cooling process.

The aim of the present report is to systematically study the isothermal and non-isothermal cold crystallization kinetics of PA12 powder in the SLS process. On the one hand, the process is similar to a quasi-isothermal process at the temperature of the building chamber at about 168–172 °C [7]. On the other hand, the isothermal crystallization at this temperature cannot be detected since the signal is too small to measure. The isothermal crystallization near the building chamber temperature was measured to understand the internal process. Also, there is a small cooling rate in the z-direction that depends on the energy input of the building parts (size, build parameters, etc.) [8]. Different positions in the building chamber also have different cooling rates. Therefore, the cooling rate for the non-isothermal crystallization is given in a range between 0.2 and 20 K/min. In the end, a comparison between the laser sintering part and isothermal crystallization analysis will be shown.

A literature review yields several reports that describe modeling for PA12 [9,10,11,12]. However, the literature on the comparison among different non-isothermal models has not been presented. Moreover, the relative deviations between measurements and modeling have not been shown.

### 1.1. Isothermal Crystallization Kinetic Model

Crystallization is usually treated as a two-stage process: the primary crystallization stage and the secondary crystallization stage [13]. Isothermal crystallization of a polymer is frequently characterized by the induction time and the crystallization halftime. More detailed analysis of isothermal crystallization is usually based on the Avrami theory [14,15,16]:

(1) $$X\left(t\right)=1-\exp\left(-k\left(T\right)t^{n}\right)$$

where the Avrami exponent n stands for the (homogeny or heterogenic) nucleation mode and dimensionality of crystal growth [17]; the crystallization rate parameter k(T) is temperature dependent. The isothermal crystallization kinetic is the base of non-isothermal crystallization kinetics. The values of the Avrami exponent are often used in non-isothermal crystallization kinetics.

To determine the Avrami exponent n and the values of the crystallization rate parameters k from the slope of the straight line and the ordinate axis intersection, Equation (1) yields:

(2) ln(−ln(1−X(t)))=lnk(T)+nlnt

Details of isothermal crystallization can be found in Section 3.2.

### 1.2. Non-Isothermal Crystallization Kinetic Model

Ozawa extended the Avrami theory to be able to describe the non-isothermal crystallization as well [18]. The degree of crystallization can be written as a function of the heating/cooling rate Φ according to the following equation:

(3) $$X\left(t\right)=1-\exp\left(-k\left(T\right)\varphi^{m}\right)$$

where k(T) is the Ozawa crystallization rate constant and m is the Ozawa exponent (which is similar to the Avrami exponent). Usually, a plot of ln(1 − ln(1 − X(t))) versus lnΦ at a given temperature is given. A straight line should be obtained if the Ozawa method is valid [19]. However, the cooling rate range used by Ozawa for the analysis of PET kinetics was from 1 to 4 K/min, which is much smaller than the range in this study [18].

Considering the non-isothermal character of the process investigated, Jeziorny suggested that the value of the rate parameter K_c_ should be adequately corrected [20] to where the crystallization rate parameter K_c_ refers to the heating/cooling rate value Φ. The K_c_ parameter does not have the same physical meaning as in the isothermal crystallization because of the constant temperature changes in non-isothermal crystallization:

(4) $$\ln K_{C}=\frac{\ln K}{\varphi }$$

In selective laser sintering, there is no constant cooling rate. Therefore, these models are not suitable for crystallization modeling in this process. Applying more general non-isothermal conditions, Nakamura developed a model as [21,22,23]:

(5) $$X\left(t\right)=1-\exp\left(-{\left(\int_{0}^{t}K\left(T\right)d\tau \right)}^{n}\right)$$

The parameter n is equal to the Avrami exponent and K(T) is a modification of the isothermal crystallization rate parameter of the Avrami rate k(T). Mathematically, they deviate from the following equation:

(6) $$K\left(T\right)=k{\left(T\right)}^{\frac{1}{n}}=\ln{\left(2\right)}^{\frac{1}{n}}\left(\frac{1}{t_{1/2}}\right)$$

where n and t_1/2_ are temperature dependent. Ziabicki proposed an empirical equation using the Lauritzen–Hoffman theory for the temperature dependency of crystallization halftime t_1/2_ as follows [24,25]:

(7) $$\left(\frac{1}{t_{1/2}}\right)=K_{0}\exp\left(-\frac{U^{*}}{R(T-T_{\infty })}\right)\exp\left(\frac{K_{g}\left(T+T_{m}^{0}\right)}{2T^{2}\Delta T}\right)$$

where K_0_ is the growth rate constant; U* = 6270 J/mol is the activation energy for polymer diffusion; R = 8.314 J/mol·K is the universal gas constant; T_∞_ = T_g_ − 30 K; K_g_ is the nucleation rate constant; ΔT = T_m_^0^ − T and T_m_^0^ is the equilibrium melting temperature [24].

To use the Lauritzen–Hoffman theory, the constants K_0_ and K_g_ from isothermal measurements are determined using temperature-dependent t_1/2_.

(8) $$\ln\left(\frac{1}{t_{1/2}}\right)+\frac{U^{*}}{R(T-T{}_{\infty })}=\ln K_{0}-\frac{K_{g}\left(T+T_{m}^{0}\right)}{2T^{2}\Delta T}$$

In this case, the constant K_0_ and K_g_ will be used in the Nakamura model in Equation (5) to model the non-isothermal crystallization and the modified crystallization rate parameter K(T) from Equation (6) would be varied to give the following Equation (9):

(9) $$K\left(T\right)={\left(\ln2\right)}^{\frac{1}{n}}K_{0}\exp\left(-\frac{U^{*}}{R\left(T-T_{\infty }\right)}\right)\exp\left(\frac{K_{g}\left(T+T_{m}^{0}\right)}{2T^{2}\Delta T}\right)$$

In the end, deviation analysis of time-X(t)-curves and temperature-X(t)-curves is used to understand the model better. The relative deviation is described at each temperature by:

(10) $$\frac{t_{\mathrm{mod}el}-t_{\mathrm{measurement}}}{t_{\mathrm{measurement}}}\cdot 100$$

## 2. Materials and Methods

The experiments were performed using PA12 powder (type: PA 2200, supplier: EOS). The particle size analysis was carried out using a particle size analyzer Morphologi G3s from Malvern Instruments. The particle diameters volumetrically are as follows: d_50,3_ is 61 µm, d_10,3_ is 46 µm, and d_90,3_ is 81 µm. The viscosity of the melt at 200 °C is 2616 ± 99 Pa·s. [26]

Isothermal and non-isothermal crystallization kinetics were carried out using a differential scanning calorimeter Q2000 from TA Instruments. The DSC was carried out under nitrogen atmosphere and the sample weights were between 2.5 and 3.5 mg.

Since the used material is highly temperature-sensitive, polycondensation accrued at high temperatures for long heating times. In order to avoid this effect, the samples were heated quickly above the melting temperature and then held there briefly. ISO 11357-7:2015 “Plastics—Differential scanning calorimetry (DSC)—Part 7: Determination of crystallization kinetics” was used.

**Figure 1:**
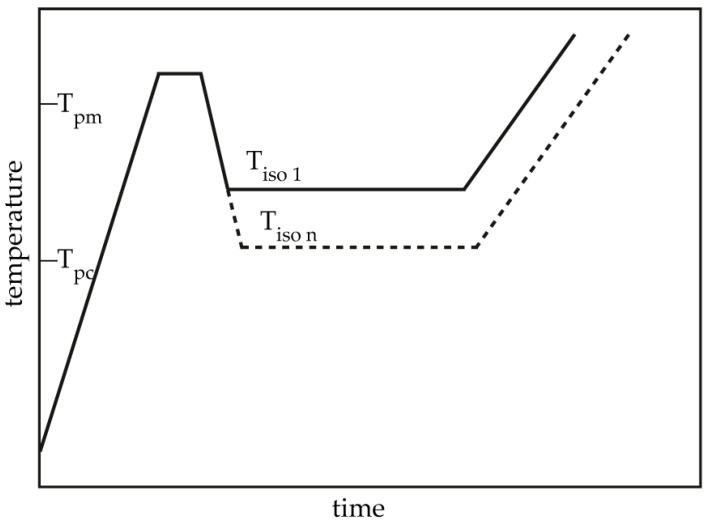
Schematic temperature program used in isothermal crystallization differential scanning calorimetry (DSC) measurements.

The isothermal crystallization process was performed as follows as in Figure 1: The samples were heated at 60 K/min to 200 °C, held for 1 min to eliminate residual crystals, then cooled at −60 K/min to the designated crystallization temperature (T_c_) in the range of 160–168 °C for isothermal crystallization for 120 min. Then, the samples were heated to 215 °C at a rate of 10 K/min to determine the polymer crystallization behavior during isothermal crystallization.

The non-isothermal crystallization was performed as follows: The samples were heated at 60 K/min to 200 °C and held for 1 min to eliminate residual crystals. Then, the melt was cooled at −60 K/min to 180 °C and after that at different cooling rates: 0.2, 0.5, 1, 2, 5, 10, and 20 K/min. The exothermic curves of heat flow as a function of time and temperature were recorded and investigated.

An experimental comparison of the SLS process was set as follow: The samples were produced at a building temperature at 172 °C. The laser power P_L_ was 19.8 W and the scanning speed v_s_ was 2475 mm/s. The laser hatching h_L_ of 0.2 mm and the layer thickness of 0.1 mm were set as production parameters. The calculated volume energy density (E_D_ = P_L_/(h·v_s_·d_s_)) was 0.4 J/mm^3^. The sample was taken into the DSC with a heating rate of 10 K/min to 200 °C to determine the polymer crystallization behavior during the SLS process.

## 3. Results and Discussion

### 3.1. Melting Behavior and Equilibrium Melting Temperature

**Figure 2:**
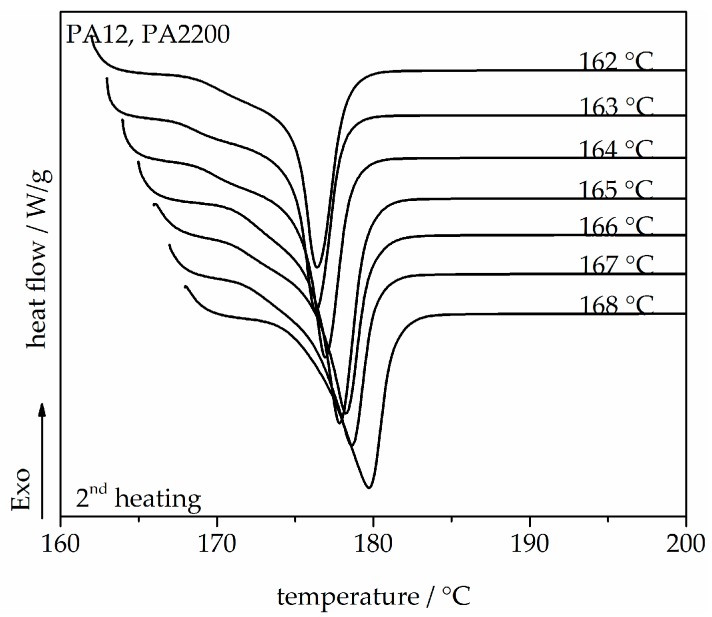
Melting endotherms of PA12, recorded at a heating rate of 10 K/min, after isothermal crystallization for the shown temperatures.

A series of DSC heating thermograms of PA12 samples are shown in Figure 2, which exhibit the second heating and shows that the melt crystallized at different T_c_ between 162 and 168 °C. One melting peak mainly refers to the melting of one crystal structure. It is well shown that the melting temperature depends on the crystallization temperature. With increasing isothermal crystallization temperature, the peak melting temperature also increases from 176.4 to 179.8 °C since bigger and more perfect crystals are expected in the region where diffusion predominates (high T_c_) [27,28]. The equilibrium melting temperature T_m_^0^ of a polymer crystal is defined as the melting temperature of an extended chain crystal and is one of its most important thermodynamic properties. T_m_^0^ would be used for non-isothermal modeling.

**Figure 3:**
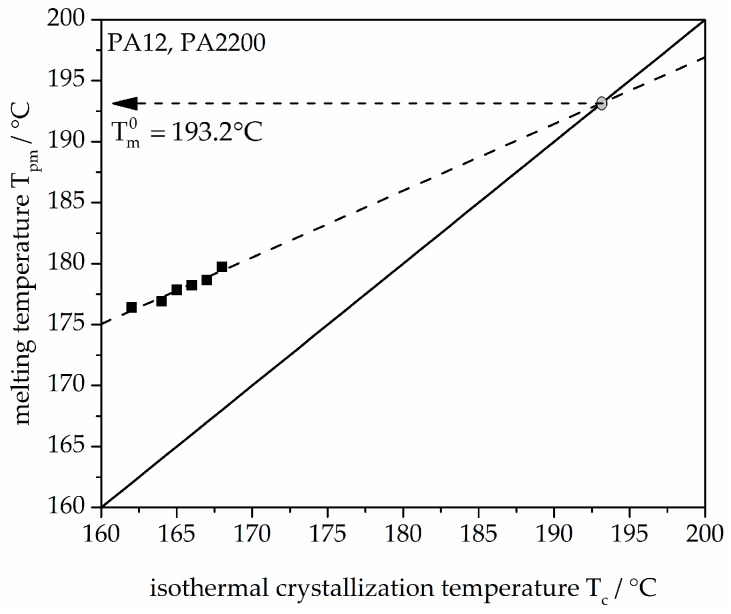
Melting temperature as a function of crystallization temperature for the PA12 samples.

Hoffman–Weeks theory is based on the difference between melting temperature, T_m_, and crystallization temperature, T_c_. Figure 3 shows the plot of T_pm_ versus T_c_. T_pm_ is the melting peak temperature. There is a linear relationship (dashed line) of experimental data (black dots) of T_pm_ and T_c_. The measured values were linearly extrapolated with the solid straight line (T_pm_ = T_c_). The intersection with the extrapolated line characterizes the equilibrium melting temperature T_m_^0^ of PA12 (PA2200), which is 193.2 °C. A comparable temperature from Amado [10] is 192.4 °C.

### 3.2. Isothermal Crystallization Kinetics Analysis

**Figure 4:**
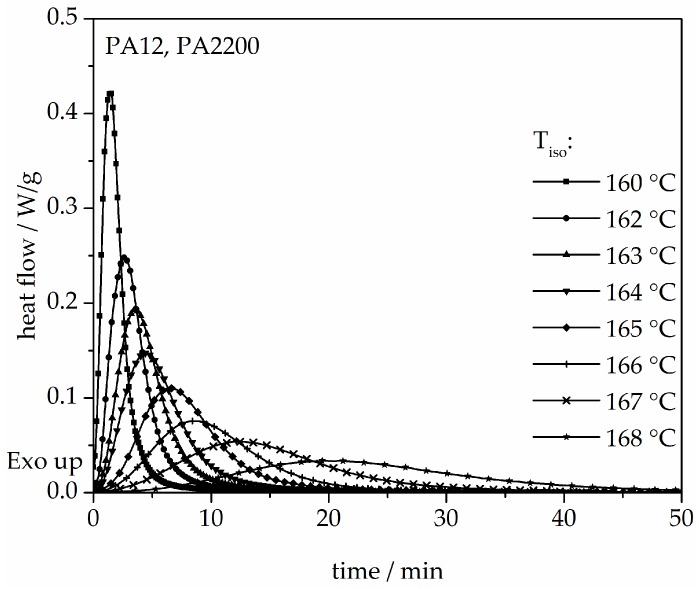
Heat flow versus time during isothermal crystallization of PA12 at different temperatures by DSC.

**Figure 5:**
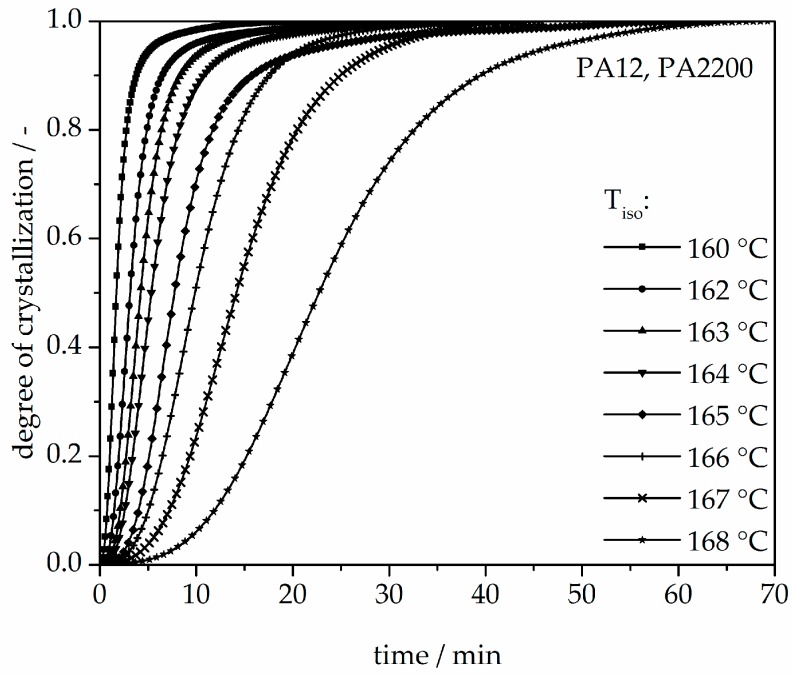
Degree of crystallization at different crystallization temperatures in the process of isothermal crystallization of PA12.

The isothermal crystallization results obtained for the isothermal measurements between 160 and 168 °C of PA12 are depicted in Figure 4. It can be observed that as the isothermal temperature decreases, the time at which the signal reaches its maximum is considerably shorter. The results of the calculated crystallization halftime t_1/2_ can be acquired from the curves depicting the degree of crystallization (see Figure 5 and Table 1). All curves have a sigmoidal course with a decreasing turnover speed at rising measured temperature. According to isothermal measurements, the depiction of the relative conversion of crystallization over time shows similar behavior. As the holding temperature increases, the crystallization proceeds more slowly. Normally, we understand a two-phase mixed state (powdery and molten) during the building process since SLS is a quasi-isothermal process at the building chamber temperature. However, in isothermal crystallization, we found that the sample would already be crystallized after 50 min at a temperature of 168 °C. This indicates that the components in the lower layers crystallize during the building process.

**Figure 6:**
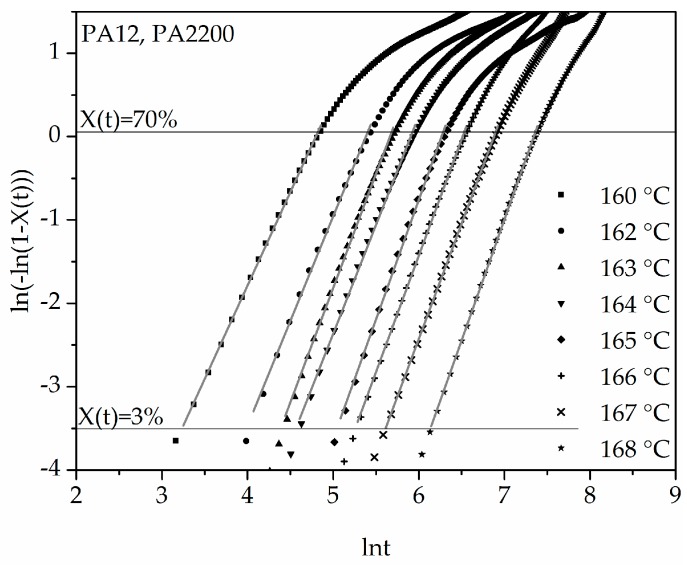
Plot of ln(−ln(1 − X(t))) versus ln t for isothermal crystallization at the indicated temperatures.

The double logarithmic plot of ln(−ln(1 − X(t))) versus ln(t) from Equation (7) is shown in Figure 6. It can be inferred from the applications that good linearization (gray curves) is possible for a degree of crystallization between 3% and 70%. The results of n and k are listed in Table 1. It is important in the calculation to achieve the highest possible accuracy of the results, which is why the “linear fit” should have at least one determination measure R^2^ > 0.99. The Avrami exponent n and the crystallization rate parameters are temperature dependent. With increasing temperature, the values of n increase and the values of k decrease. The Avrami exponent varies between 2.24 and 2.9, which means that crystal growth is a mixture of 2D and 3D. For the polyamides that tend to be recrystallized, several linear curves should be fixed [12].

**Figure 7:**
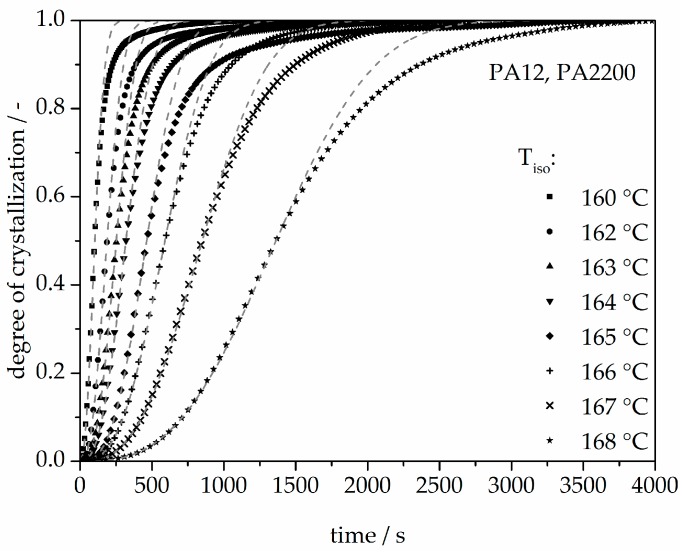
Isothermal crystallization kinetic comparison between the measurement and the modeling for the PA12.

**Figure 8:**
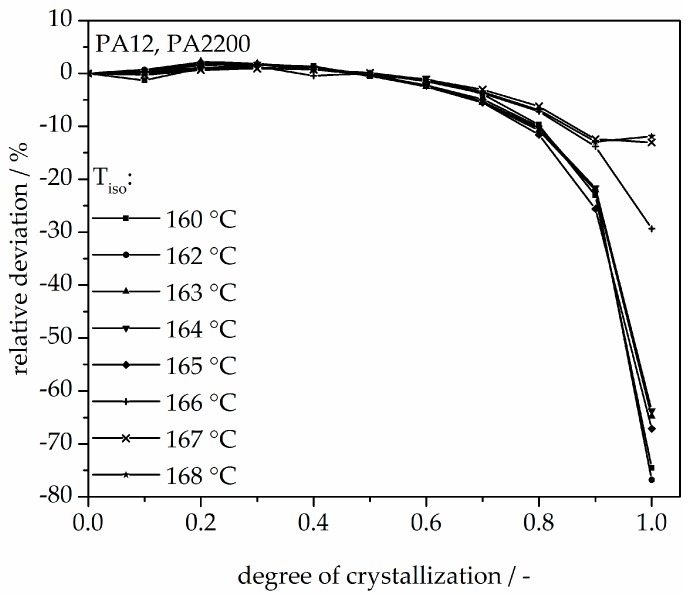
Deviation analysis of time-X(t)-curve at different temperatures.

The calculated curves for the degree of crystallization are shown in Figure 7 together with the experimental values. The curves fit together very well at the beginning. The crystallization halftime t_1/2_ from the modeling curves depicting the degree of crystallization is also shown in Table 1. A list of n and k from [11] is also given in Table 1. The n value matches with this study but there are no units for k used in the literature. There are minimal differences in the results of t_1/2_ between the measurements and the model. However, the results of modeling after crystallization rates of 70% and higher differ from the measurements. Because the “linear fit” only follows the degree of crystallization up to 70%, a deviation analysis of time-X(t)-curves was used to understand the model better. The relative deviation from Equation (8) is shown in Figure 8. A small deviation (<±5%) in the measurement and modeling are seen before X(t) ≤ 70%. The relative deviation rises with sinking isothermal temperature. There is great variance up to even 80% at 160 °C isothermal crystallization. The reason for the deviations are the continued growth of the spherulite boundaries and the transformation of the internal structure in a spherulite.

As shown in state of the art, the building chamber temperature is usually set at 172 °C or higher but measurements are no longer performed in the DSC over 168 °C. Therefore, the isothermal crystallization experiments were carried out at lower temperatures. Then, they were compared with the models and evaluated so that modeling could be applied to depict the process at higher temperatures.

### 3.3. Non-Isothermal Crystallization Kinetic Analysis

**Figure 9:**
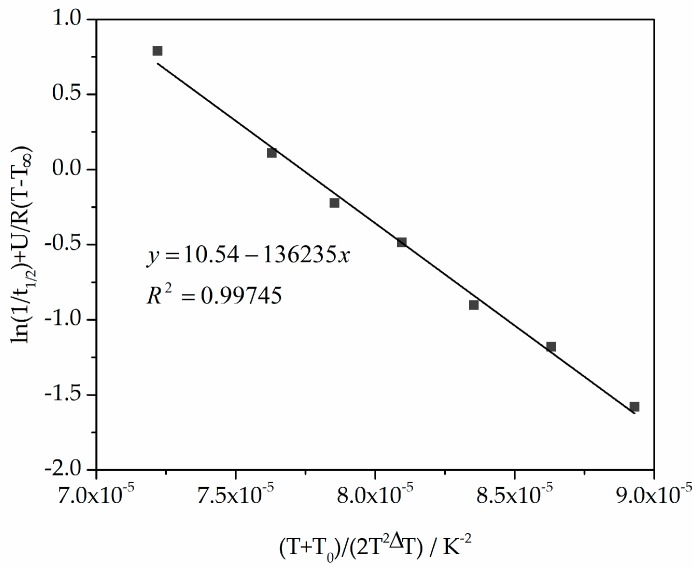
Plot of ln(1/t_1/2_) + U/R(T − T_∞)_ versus (T + T_0_)/(2T^2^ΔT) for calculating the parameters K_0_ and K_g_ used in the Hoffman–Lauritzen theory.

To use the Lauritzen–Hoffman theory, the constants K_0_ and K_g_ from isothermal measurements were determined using temperature-dependent t_1/2_ , as shown in Table 1 [29], and from Equation (8) the straight line slope and the ordinate axis intersection. The double logarithmic plot of ln(1/t_1/2_) + U/R(T − T_∞_) versus (T + T_0_)/(2T^2^ΔT) is shown in Figure 9.

From Figure 9 and Equation (8), the constants K_0_ and K_g_ are 37,869 s^−1^ and 136,235 K^2^. The comparable values from Amado [10] are 55,600 s^−1^ and 140,866 K^2^.

**Figure 10:**
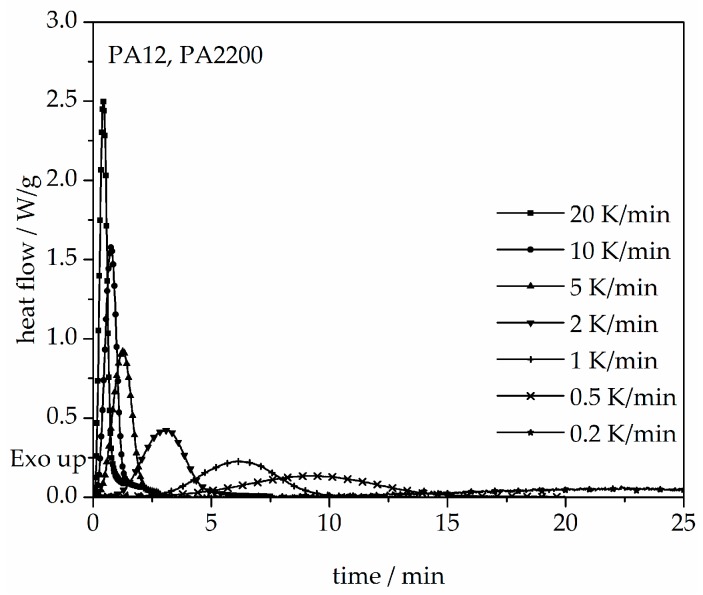
Heat flow versus time during non-isothermal crystallization of PA12 at different cooling rates by DSC.

The results obtained for the non-isothermal measurements between 20 and 0.2 K/min of PA12 are depicted in Figure 10. From the original data, the crystallization onset temperature needed to be interpreted for the modeling. It can be observed that a slower cooling rate leads to a longer time at which the signal reaches a maximum t_max_ and shifts the crystallization onset temperature T_ic_ toward higher values. Values for t_max_ and T_ic_ are shown in Table 2. In addition, the results for the calculated crystallization halftime t_1/2_ are also included in Table 2.

**Figure 11:**
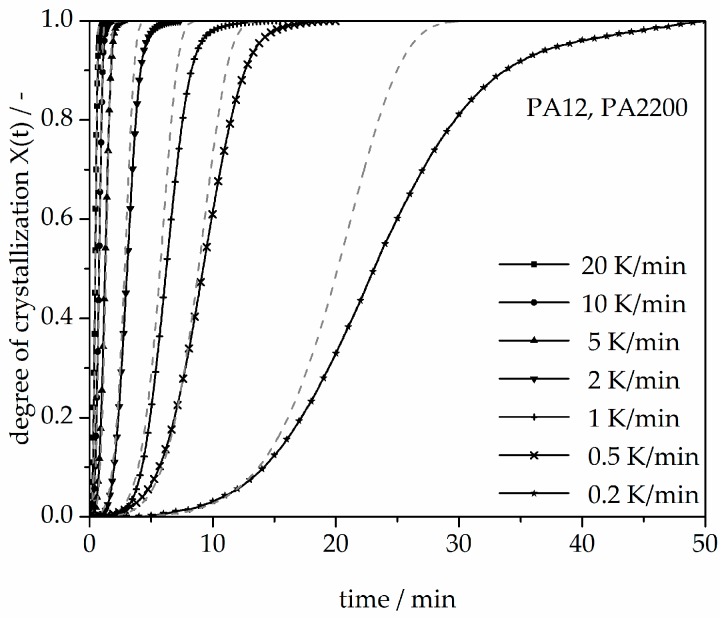
Non-isothermal crystallization kinetic comparison between the measurements and the modeling for the PA12 plot of time.

**Figure 12:**
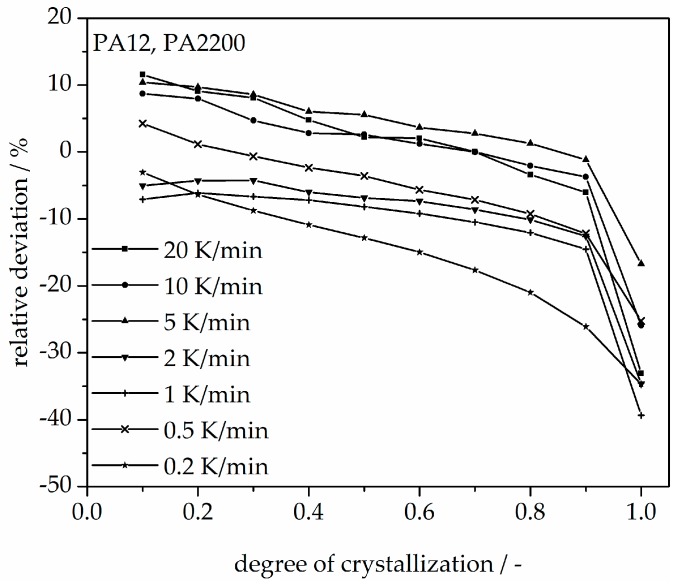
Deviation analysis of time-X(t)-curve at different cooling rates.

A non-isothermal crystallization kinetic comparison between the measurements and the modeling for the PA12 plot of time is shown in Figure 11. The curves at different cooling rates for the modeling and measurements are the same. The modeled degree of crystallization curves are slower than those measured. To explain the relative deviation, a deviation analysis using Equation (8) was performed (see Figure 12). The relative deviations of measurement and modeling at 0.5, 1, 2, 5, 10, and 20 K/min up to X(t) = 0.9 are within ±10%. On the one hand, for cooling rates of 5, 10, and 20 K/min, the deviations are plus values, meaning that the modeling values are larger than the measurements. On the other hand, for cooling rates of 1 and 2 K/min, the values are negative. The largest deviation is the curve at 0.2 K/min. Because of the unstable measurement signal at this cooling rate, the relative deviation is high. In summary, the relative deviation at X(t) = 1 would reach up to 40%. The cooling rate in the process is dependent on the height of the building job and the position of the parts. To understand the crystallization behavior of the parts, we need the temperature gradient in the building parts during a building job. The results of the calculated crystallization halftime t_1/2_ from the modeling are listed in Table 2.

**Figure 13:**
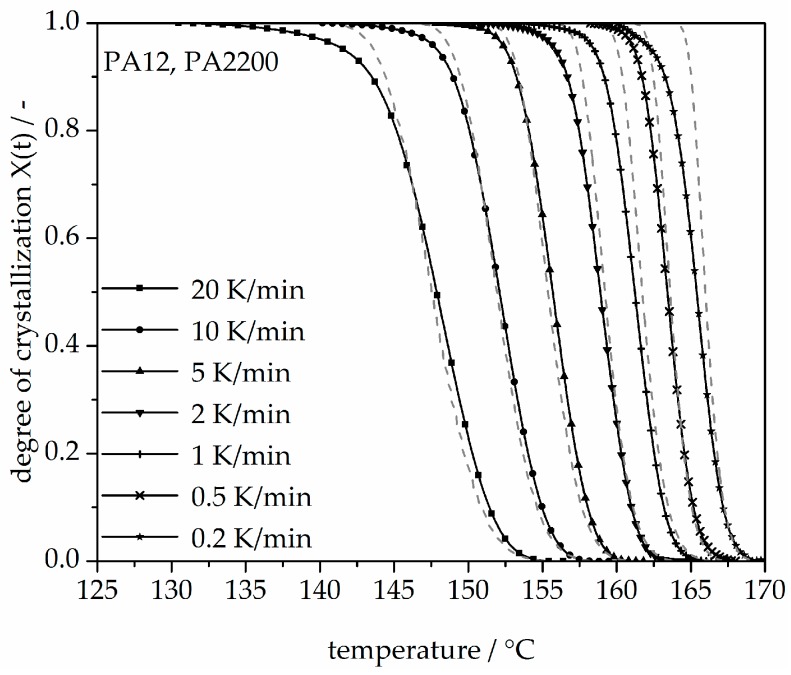
Non-isothermal crystallization kinetic comparison between the measurement and the modeling for the PA12 plot of temperature.

**Figure 14:**
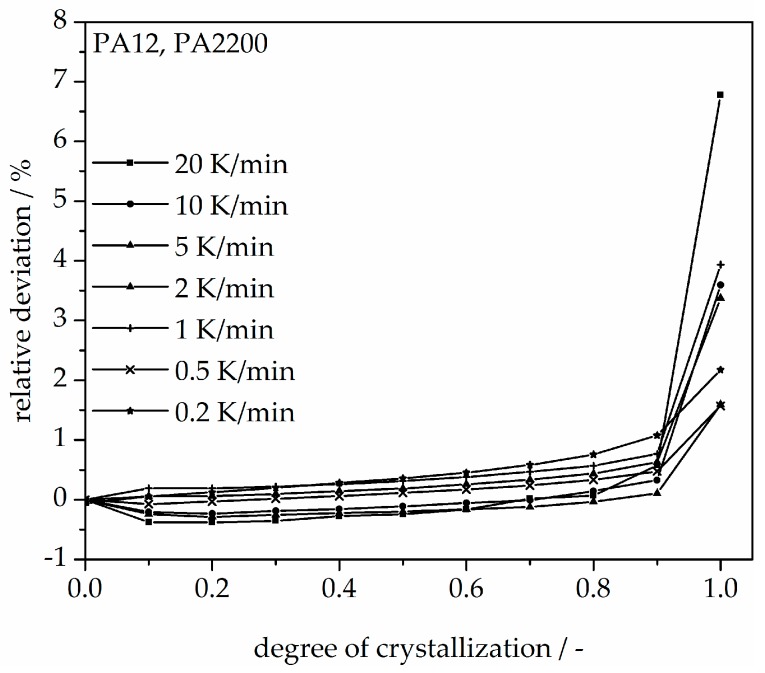
Deviation analysis of temperature-X(t)-curve at different cooling rates.

In addition, changing the cooling rate changes not only the crystallization time but also the crystallization temperature. As was shown at the beginning of this section, the crystallization onset temperature T_ic_ needs to be applied to the modeling. In Figure 13, a non-isothermal crystallization kinetic comparison between the measurement and the modeling of the PA12 plot for temperature is depicted. Comparing to the plot of time, slower cooling rates lead to narrow temperature ranges. The deviation analysis of temperature-X(t)-curve at different cooling rates is shown in Figure 14. It allows only ±1% with a minimum deviation of all the curves up to X(t) < 0.9.

With a 0.2 K/min cooling rate, the crystallization begins at 170 °C, then runs to 160 °C. At the 20 K/min stirring, the initial crystallization temperature is 155 °C, which means that the crystallization temperature depends on the cooling rate. In the process, the building chamber was set at 172 °C and the removal temperature at 150 °C. In this temperature range, the material crystallizes.

**Figure 15:**
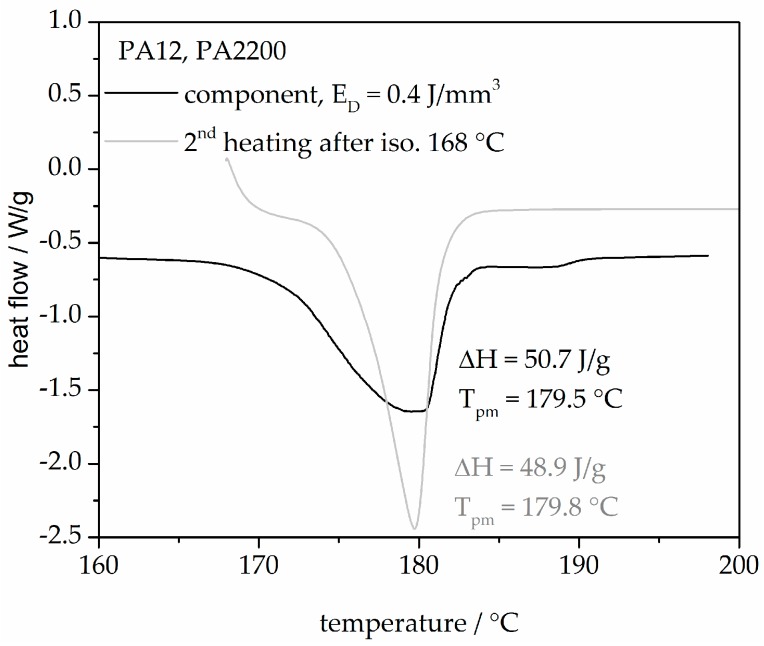
A comparison between a second heating of isothermal crystallization at 168 °C and the first heating of a laser-sintered component.

Figure 15 shows two heating curves from an SLS component built with 0.4 J/mm^3^ and a second heating after isothermal crystallization at 168 °C for 120 min. The heating curve of the component describes the state of the sample through the thermal and mechanical history (process influences, the degree of crystallization etc.). A good agreement between the melting peak temperature and the heat of fusion from both curves shows that the modeling is very helpful for understanding the process.

## 4. Conclusions

Applying crystallization kinetics in the SLS process is a new approach for process-adapted characterization of plastics since crystallization kinetic studies are used mostly for crystallization processes in solutions. This paper presents a crystallization kinetics study via the selective laser sintering process. Based on isothermal and non-isothermal crystallization measurements, the crystallization kinetics were determined. The higher the isothermal holding temperature, the longer the crystallization takes. In addition, with a smaller cooling rate, the initial temperature is higher and the crystallization time is longer. For isothermal crystallization, a small deviation (<±5%) between the measurement and the modeling is seen before X(t) ≤ 70%. For non-isothermal crystallization, the relative deviations plot of time between measurement and modeling at 0.5, 1, 2, 5, 10, and 20 K/min up to X(t) = 0.9 are within ±10%. A minimum deviation plot of the temperature of all the curves up to X(t) < 0.9 is only ± 1%. A good agreement between the heat flow curves from an SLS component and the second heating after isothermal crystallization at 168 °C shows that the modeling is very helpful for understanding the process. Knowing the crystallization kinetics in combination with the temperature profile of a component during laser sintering will allow prediction of the local crystallization behavior in dependency of position. According to state of the art, the temperature history of a component after exposure, during laser sintering, and in the cooldown stage cannot be measured. Therefore, the thermal history will be simulated in further research. The resulting temperature profile builds the basis of calculation of the resulting degree of crystallization.

## Figures and Tables

**Table 1:** Kinetic parameters for isothermal crystallization of PA12.

T_c_ °C	n	n [11]	k	k [11]	t_max_	t_1/2_ Measurement	t_1/2_ Model
	-	-	S^−1^		s	s	s
160	2.24	2.71	2.15 × 10^−5^	2.65 × 10^−7^	88.6	103.2	102.6
162	2.51	2.94	1.37 × 10^−6^	1.34 × 10^−8^	158.4	188.4	187.8
163	2.74		1.81 × 10^−7^		212.4	253.3	252.0
164	2.61	3.05	2.01 × 10^−7^	1.08 × 10^−9^	260.4	319.8	319.2
165	2.87		1.59 × 10^−8^		396.6	463.8	462.6
166	2.79	2.69	1.28 × 10^−8^	2.62 × 10^−9^	510.0	591.0	591.0
167	2.77		5.44 × 10^−9^		738.6	849.0	849.6
168	2.90	2.94	5.69 × 10^−10^	6.34 × 10^−11^	1179.0	1362.6	1362.6

**Table 2:** Kinetic parameters for non-isothermal crystallization of PA12.

Φ K/min	T_ic_	t_max_	t_1/2_ Measurement	t_1/2_ Model
	°C	s	s	s
20	156.4	25.8	27.6	28.2
10	169.5	45.0	46.2	47.4
5	161.8	75.0	75.6	79.8
2	164.9	186.6	183.0	170.4
1	167.4	367.8	373.2	342.6
0.5	168.0	558.6	553.2	533.4
0.2	170.0	1341.0	1387.2	1208.4
